# Melt segregation from silicic crystal mushes: a critical appraisal of possible mechanisms and their microstructural record

**DOI:** 10.1007/s00410-018-1465-2

**Published:** 2018-05-23

**Authors:** Marian B. Holness

**Affiliations:** Department of Earth Sciences, University of Cambridge, Downing Street, Cambridge, CB2 3EQ UK

**Keywords:** Rhyolite, Melt segregation, Crystal mush, Compaction, Settling

## Abstract

One of the outstanding problems in understanding the behavior of intermediate-to-silicic magmatic systems is the mechanism(s) by which large volumes of crystal-poor rhyolite can be extracted from crystal-rich mushy storage zones in the mid-deep crust. The mechanisms commonly invoked are hindered settling, micro-settling, and compaction. The concept of micro-settling involves extraction of grains from a crystal framework during Ostwald ripening and has been shown to be non-viable in the metallic systems for which it was originally proposed. Micro-settling is also likely to be insignificant in silicic mushes, because ripening rates are slow for quartz and plagioclase, contact areas between grains in a crystal mush are likely to be large, and abundant low-angle grain boundaries promote grain coalescence rather than ripening. Published calculations of melt segregation rates by hindered settling (Stokes settling in a crystal-rich system) neglect all but fluid dynamical interactions between particles. Because tabular silicate minerals are likely to form open, mechanically coherent, frameworks at porosities as high as ~ 75%, settling of single crystals is only likely in very melt-rich systems. Gravitationally-driven viscous compaction requires deformation of crystals by either dissolution–reprecipitation or dislocation creep. There is, as yet, no reported microstructural evidence of extensive, syn-magmatic, internally-generated, viscous deformation in fully solidified silicic plutonic rocks. If subsequent directed searches do not reveal clear evidence for internally-generated buoyancy-driven melt segregation processes, it is likely that other factors, such as rejuvenation by magma replenishment, gas filter-pressing, or externally-imposed stress during regional deformation, are required to segregate large volumes of crystal-poor rhyolitic liquids from crustal mushy zones.

## Introduction

One of the outstanding problems in understanding the behavior of intermediate-to-silicic magmatic systems is the processes(s) by which voluminous eruptions of crystal-poor evolved liquid can be generated (Lundstrom and Glazner 2016). Suggestions range from efficient segregation of liquid from a partially molten source in the deep crust (e.g., Clemens and Stevens 2016), to extraction of interstitial liquid from silicic crystal mushes in the mid-deep crust (Hildreth 2004). This contribution is a critical appraisal of the latter suggestion, with a focus on the mechanisms by which liquid might segregate from a crystal mush.

In a highly influential paper, Bachmann and Bergantz (2004) addressed the problem of time-scales of segregation of crystal-poor liquid from a crystal mush by considering possible physical mechanisms by which solids and liquids could separate from each other under conditions in which the crystal content was sufficiently high, estimated to be ~ 40 vol.%, that the magma was unable to flow (i.e., surpassing the Rigid Percolation Threshold of Vigneresse et al. 1996). They suggested that segregation time-scales could be constrained by a consideration of two end-member mechanisms: first, gravitational settling of individual solid grains through a liquid containing sufficiently abundant crystals that simple Stokes’ settling calculations are no longer valid (this is known as hindered settling); and second, expulsion of interstitial liquid during gravitationally-driven compaction of the crystal matrix. The latter process, involving plastic deformation of solids, is here termed viscous compaction to differentiate it from the mechanical compaction which occurs when essentially rigid, non-deforming grains are re-arranged by, e.g., magmatic currents, slumping of the mushy layer, internally generated pressure gradients caused by de-gassing, or externally generated syn-magmatic stress fields. Hindered settling was considered by Bachmann and Bergantz (2004) to represent the fastest mechanism for melt segregation, while viscous compaction represents the slowest end-member. A third mechanism, operating at intermediate rates, was suggested to be the micro-settling process first described in the geological literature by Miller et al. (1988).

Importantly, Bachmann and Bergantz (2004) do not appear to have intended to present a definitive identification of the operative mechanisms driving melt segregation from crystal mushes: they used well-established analytical treatments of hindered settling and viscous compaction purely to place constraints on the time-scales of melt segregation. However, many subsequent studies (e.g., Ellis et al. 2014; Bachmann and Huber 2016; Lee et al. 2016) have tended to list the three Bachmann and Bergantz (2004) mechanisms as though they are, indeed, dominant during segregation of silicic melts, with little critical assessment of their relative importance and no attempt to test whether any unambiguous evidence of their operation is preserved in the rock record. The first part of this contribution is a review of the physical basis for these processes, with a discussion of their microstructural record. The second part comprises a discussion of the role which other factors might play in melt extraction from crystal mushes.

## Micro-settling

**Fig. 1:**
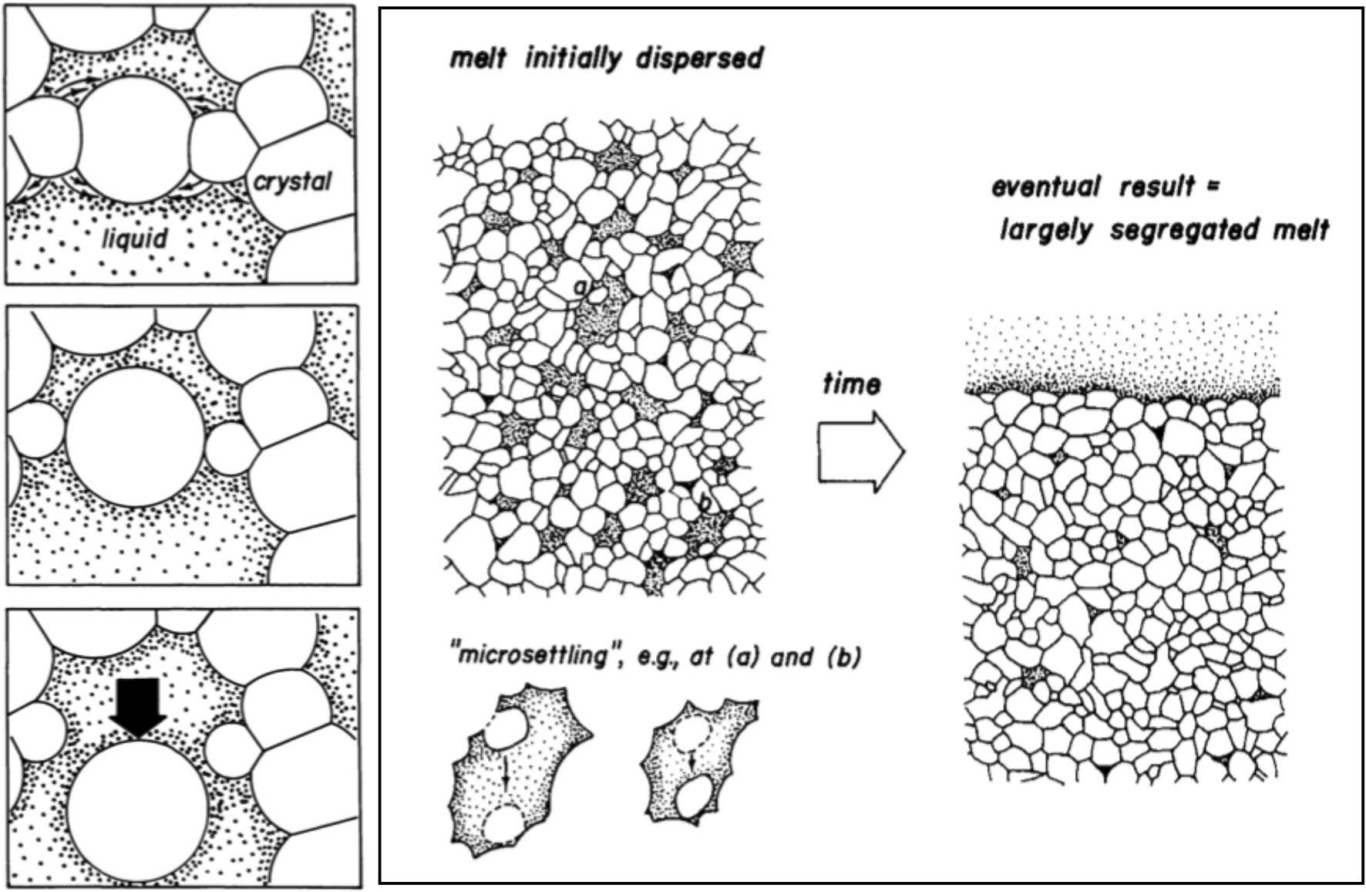
Cartoons showing the operation of micro-settling, as initially proposed by Niemi and Courtney (1983). Reproduced, by permission of The Royal Society of Edinburgh and EB Watson, from Miller et al. (1988)

Miller et al. (1988) suggested, following a study published by Niemi and Courtney in 1983, that a process which they called micro-settling might play a role in melt segregation in silicic systems. Micro-settling is envisaged as a form of gravitationally-driven compaction that does not require, or result in, viscous deformation. It involves the detachment of individual grains within a porous, open, crystal framework and their migration under gravity to the bottom of the melt-filled pore in which they find themselves (Fig. 1). The detachment was suggested to occur as a result of Ostwald ripening (Niemi and Courtney 1983; Miller et al. 1988), a coarsening process driven by the differences in solubility as a function of grain size and surface curvature: large grains grow at the expense of small grains. Miller et al. (1988) stated that nothing was known at the time about the detachment kinetics or the rates at which melt might segregate by this process: no attempts by the geological community to rectify this have been published.

The study of Niemi and Courtney (1983) concerns the effects of gravity on the behavior of liquid-particle mixtures during liquid-phase sintering or LPS. Liquid-phase sintering is a process during which a mush densifies as the capillary forces exerted by the liquid pulls grains together, coupled with grain coalescence: it is a dissolution–reprecipitation process—see German et al. (2009) for a comprehensive and accessible review. Niemi and Courtney (1983) argued, from observations of LPS in Fe–Cu alloys, that dense particles initially sink to the floor of their liquid-filled container to form an open framework, termed a skeleton, initially comprising 20 vol.% solids. The volume fraction of 20% required for skeleton formation was argued to be system-invariant. They then demonstrated that Ostwald ripening results in the extraction of particles from the skeleton by showing that particles at the free surface of a sintered compact held in contact with liquid were readily detached. Detachment was faster in the W-(Fe–Ni–Cu) system due to the lower solid–liquid dihedral angle than the Fe–Cu system, resulting in a smaller surface area of inter-grain contacts. The observation of grain detachment at a free surface was then used by Niemi and Courtney (1983) to argue for a second, much slower, stage of settling, termed skeletal settling, that reduces in importance as the packing efficiency of the sintered aggregate increases. The suggested process of skeletal settling is precisely the micro-settling of Miller et al. (1988).

### Does the Niemi and Courtney model survive scrutiny?

Given the importance of the Niemi and Courtney model to the manufacture of materials under controlled conditions, their results have been the subject of a number of detailed investigations aimed primarily at testing their conclusion that the skeleton forms at 20 vol.% regardless of the composition of either liquid or solid. Heaney et al. (1995) demonstrated that this is incorrect by examining the volume fraction of the skeleton in a range of systems with widely varying density differences between the liquid and solid phases. They showed that higher volume fractions of solids are achieved in systems containing relatively dense solids, attributing the increase in packing efficiency to the shorter time taken for particles to sink and, therefore, the shorter time available for the formation of loose clusters that prevent efficient packing. Furthermore, Liu et al. (1995) demonstrated that LPS under gravity leads to a stratigraphic gradient in packing, with denser accumulations towards the base of the container, driven by a greater rate of pressure solution by the increased gravitational loading in systems with dense particles [this is essentially the basis for models of viscous compaction used in the geological literature, e.g., McKenzie (1984)]. They showed that Niemi and Courtney’s original data are entirely consistent with the predictions of their gravitationally-driven compaction model.

Were the Niemi and Courtney model correct, the results of the Heaney et al. (1995) and Liu et al. (1995) studies would imply that the extraction of particles by Ostwald ripening is faster and more efficient, and occurs at lower porosities, for systems with a large density difference between solids and liquids. Since micro-settling, as envisaged by Niemi and Courtney (1983), is essentially driven by diffusive processes, the density difference between solid and liquid should be immaterial. Furthermore, while Niemi and Courtney (1983) demonstrated that extraction rates (as observed at a free surface) apparently scale with dihedral angle, the relationship between dihedral angle and density difference required to validate their model for skeletal settling does not exist.

Tewari et al. (1999) undertook serial sectioning of sintered aggregates of tungsten and found virtually none of the isolated grains that would be expected if micro-settling had occurred. While it might be argued that this absence simply reflects the short time following extraction before grains fall to the base of the pore to be re-attached, and, therefore, a very low likelihood of catching grains in transit, Tewari et al. (1999) also undertook serial sectioning of the same material sintered in micro-gravity and again found almost no isolated grains. Since the tungsten particles do not sink perceptibly under micro-gravity, the absence of isolated particles under such conditions demonstrates that micro-settling of individual particles must be very uncommon and cannot be responsible for measurable densification.

As a footnote, Courtney later acknowledged (Xu et al. 2002) that micro-settling by extraction driven by Ostwald ripening is not the mechanism by which skeletal settling takes place. Xu et al. (2002) pointed out that the convincing experimental confirmation of particle extraction by Ostwald ripening presented by Niemi and Courtney (1983) was limited to the free surfaces of mushy zones, which do not bear much relationship to the mushy zone interior. They accounted for the settling initially described by Niemi and Courtney (1983) to dissolution of the first-settled crystals as the system attempted to reach chemical equilibrium in the early stages of the sintering process.

### Is micro-settling important in silicate crystal mushes?

Although the discarding of the concept of micro-settling by the LPS community might be sufficient grounds on which to argue that micro-settling will not occur in any system, given the enthusiasm with which micro-settling has been embraced by Earth scientists, it is worth considering the particular case of silica-rich crystal mushes to examine the likelihood that such a process might operate on geological time-scales. An assessment of the importance of micro-settling needs to include both a consideration of the significance of Ostwald ripening in silicate systems, and a consideration of the likelihood that Ostwald ripening can lead to extraction of grains from a crystal framework. To do this, I first present a method of detecting the microstructural signature of Ostwald ripening and then discuss the likelihood that grain extraction by Ostwald ripening occurs in silicate crystal mushes.

#### How can we detect Ostwald ripening?

Many studies examining the crystal size distribution (CSD) in magmatic rocks demonstrate a deficit at small grain sizes, and this is generally attributed to Ostwald ripening (e.g., Marsh 1988; Waters and Boudreau 1996). However, there are other possible explanations for the absence of the smallest grain size fraction, including the cessation of nucleation related to the reduction either of undercooling (Higgins 1998, 1999) or of the volume fraction of remaining porosity (e.g., Cahn 1980; Putnis and Mauthe 2001; Holness et al. 2012), or the loss of small crystals by flushing with percolating magma (c.f. Druitt 1995). What is currently missing is a robust procedure for detecting the agency of Ostwald ripening using microstructural criteria other than the distribution of grain sizes: one possible way of doing this is by considering the variation of grain shape as a function of size.

The shape of a grain suspended in a liquid is governed by the kinetics of growth and the anisotropy of interfacial energy. Grains undergoing growth at rates faster than those at which the equilibrium (minimum interfacial energy) shape can be maintained will have shapes controlled predominantly by the kinetics of interfacial attachment and the relative growth rates of the different faces. Materials for which interfacial attachment is easy tend to grow as rounded grains, whereas those which grow by mechanisms involving the nucleation and spread of new layers grow as strongly facetted grains (Kirkpatrick 1975).

**Fig. 2:**
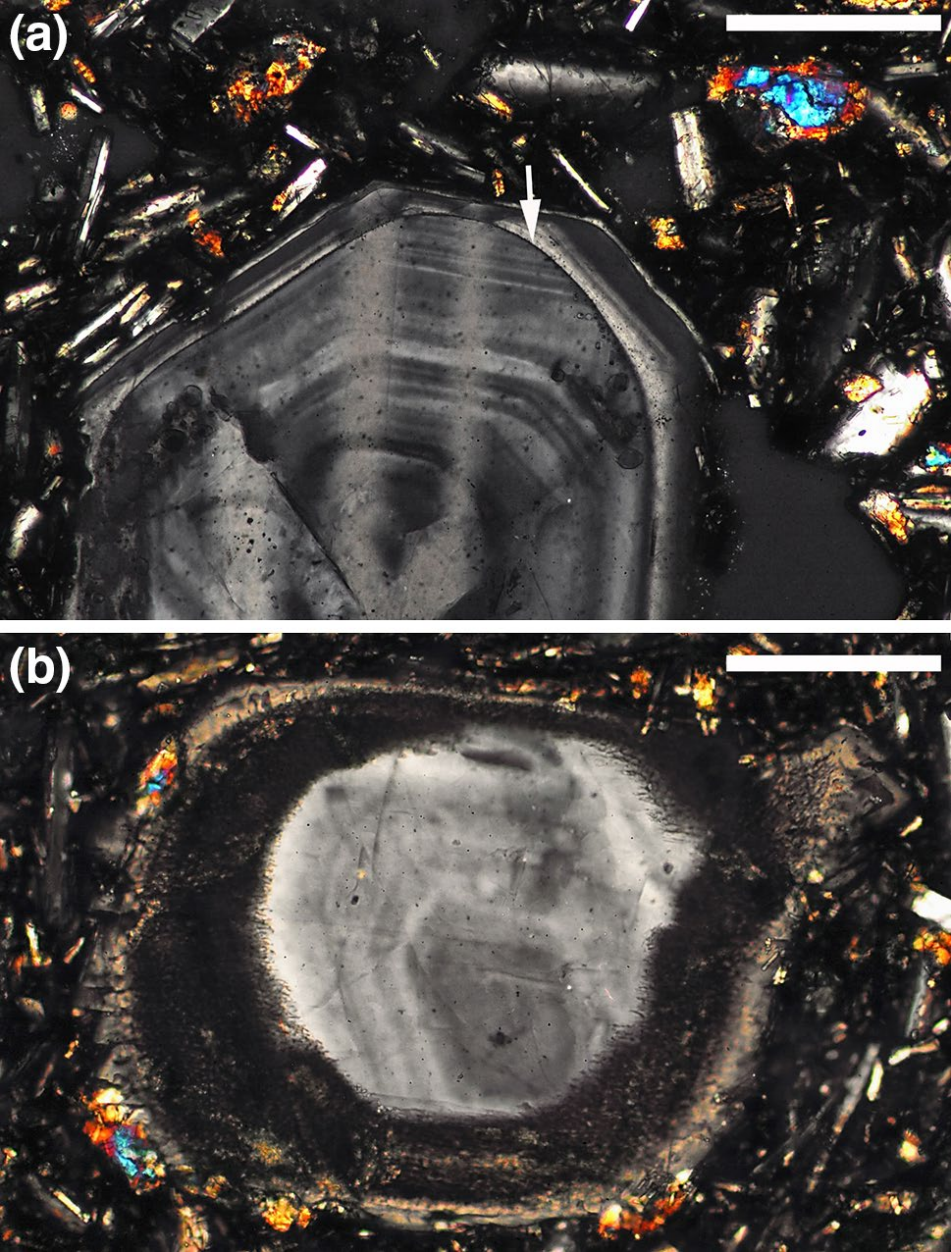
Photomicrographs under crossed polars of plagioclase phenocrysts in an andesite, Kalymnos, Greece (sample 105943 from the Harker Collection of the Sedgwick Museum, University of Cambridge). **a** Grain showing fine-scale oscillatory growth zones that have been truncated during a dissolution event (arrowed), with subsequent overgrowth re-establishing a facetted morphology. Scale bar is 200 μm long. **b** Zoned plagioclase phenocryst with incomplete dissolution marked by the outer turbid rim. Note the rounded shape of the unaffected central part of the grain, in which zoning outlines facetted growth stages. Scale bar is 200 μm long

For the specific case of a material undergoing Ostwald ripening, during which some grains grow, while others dissolve, the range in shapes depends on what is controlling dissolution and reprecipitation. If the control on both dissolution and reprecipitation is the kinetics of attachment and detachment at the fluid–solid interface, the material will comprise facetted grains, whereas if the controlling step is diffusion through the liquid (as is the case for most materials described in the LPS literature), the grains will be rounded (German et al. 2009). For silicate minerals, interface attachment kinetics are generally important during growth, leading to facetted grains that grow by the nucleation and spreading of new layers (Kirkpatrick 1975). In contrast, the kinetics of detachment during dissolution are not limited by the requirement to nucleate a new layer, as illustrated by the rounded shapes of grains undergoing dissolution and melting (Fig. 2; Scarfe et al. 1980; Watson 1982; Tsuchiyama 1985; Kuo and Kirkpatrick 1985). For Ostwald ripening in a system in which growth is controlled by interface attachment kinetics, but dissolution is not, we expect the smaller (dissolving) grains to be rounded, while the larger (growing) grains are facetted (e.g., Sarian and Weart 1965). This difference in grain shape between dissolving and growing grains provides a method of demonstrating the action of Ostwald ripening.

During Ostwald ripening, the critical radius is that separating (small) dissolving grains and (large) growing grains. In a system in which interfacial attachment kinetics is important, we would thus expect to see a round shape for all grains of radius smaller than the critical radius, while grains larger than the critical radius will have some facets. In a system in which rates of ripening are controlled by either mass transport by diffusion through the bulk matrix (e.g., a liquid), or interfacial attachment/detachment kinetics, the critical radius is the number-average radius (Wynblatt and Gjostein 1975; Finsy 2004). The critical radius in solid systems in which coarsening of particles on grain boundaries is controlled by grain boundary diffusion, of relevance to metamorphic rocks, is the harmonic average radius (Speight 1968).

**Fig. 3:**
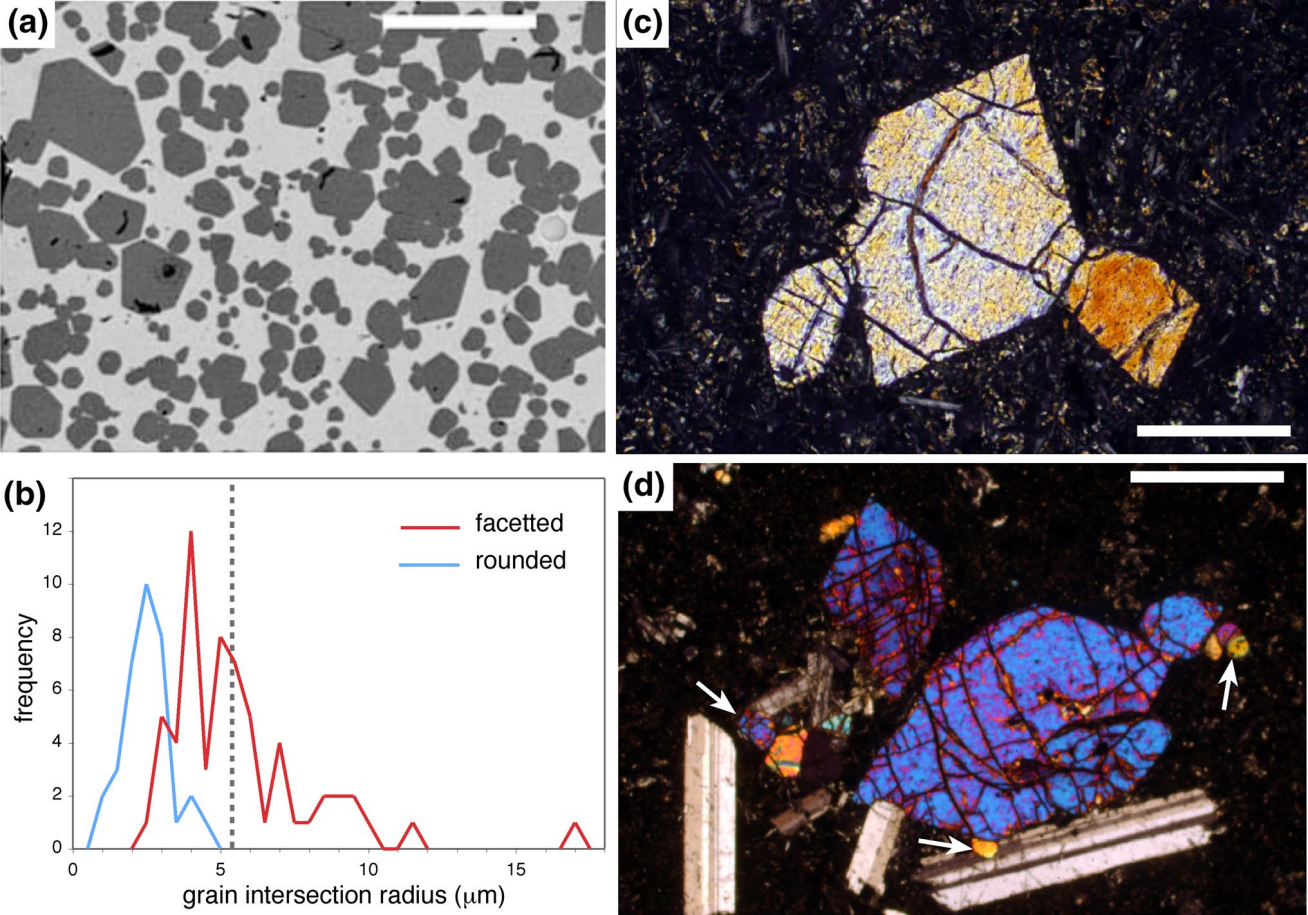
**a** Back-scatter electron image of olivine grains in quenched basaltic liquid as shown by Cabane et al. (2005) (image kindly provided by D. Laporte). The scale bar is 50 μm long. **b** Frequency plots of the radius of grain intersections, calculated from the area of each grain intersection and assuming that it is circular. The vertical dashed line gives the number-average radius of the grain intersections multiplied by a factor of 1.273 to provide the number average of 3D the grain radius. No rounded grains are larger than this number-average radius. The few facetted grains smaller than this radius are most probably intersections through larger grains. **c** Group of three strongly facetted olivine phenocrysts in the margin of a picrite dyke, Skye. The faceting supports growth of all grains. Scale bar is 200 μm long. **d** Grain cluster in the margin of a picrite dyke, Skye. The larger olivine grains have well-developed facets, whereas the smaller grains (arrowed) are rounded, suggesting some Ostwald ripening of the cluster. Scale bar is 200 μm long

During growth, olivine grains are generally bounded by some facets, demonstrating some control by interfacial attachment kinetics (e.g. Faul and Scott 2006). Using the published images of olivine grain sizes and shapes from an experimental study of Ostwald ripening of olivine (Cabane et al. 2005: their Fig. 2b is reproduced here as Fig. 3a), the sizes of olivine grains with no visible regions of planar interface can be compared with the associated grains with areas of planar facets. The effective radius for each grain was calculated assuming a circular cross-section in the image, with the frequency in each size bin, as shown in Fig. 3b. If it is assumed that all grains are spherical, the number-average radius in 3D is 1.273 times that observed in 2D (Wicksell 1925). There are no rounded grains in the image with a radius greater than that of the average true 3D grain radius (Fig. 3b), demonstrating that the critical radius in this experimental run conforms to theory. A preliminary look at olivine-phyric, rapidly quenched, magmas demonstrates that the largest grains do, indeed, tend to be facetted, while the smallest grains are rounded (Fig. 3c, d).

This simple illustration demonstrates a straightforward way to determine whether a dearth of small grains is a consequence of Ostwald ripening or whether it is the result of some other process. However, it should be remembered that grain shapes are also governed by interfacial energies: in systems kept at a constant high temperature, with the volume fraction of solids remaining essentially constant, crystal shapes are a mixture of the equilibrium shape (that with the minimum integrated interfacial energy) and growth shapes (driven by crystal growth/dissolution) (Shatov et al. 1998). In any one system, both types of shape are created by exactly the same dissolution–reprecipitation mechanism: equilibrium shapes are generated by reprecipitation on the same grain, whereas growth-controlled shapes are generated by reprecipitation on a different grain. The influence of textural equilibration means that the effects of Ostwald ripening on grain shape as a function of size will be amplified, with smaller grains having a shape closer to equilibrium, whereas larger crystals have growth-dominated shapes.

#### Is extraction by Ostwald ripening significant in a silicate mush?

The answer to this question requires us to determine first whether Ostwald ripening plays a major role in silicate crystal mushes, and secondly whether such Ostwald ripening can result in grain extraction. Critically, Ostwald ripening is most likely to be effective in systems held at approximately constant temperatures for sustained periods. Although ripening can be accelerated by temperature cycling (Simakin and Bindeman 2008; Mills et al. 2011), such cycling is unlikely in a large non-convecting crystal mushy zone [unless, of course, it is disturbed repeatedly by the arrival of new batches of magma, e.g., during incremental assembly (Coleman et al. 2004; Schoene et al. 2012)]. However, such bodies have a long-lived super-solidus history, in which the crystallinity is thought to be in the range 40–60%, promoted by the prolonged release of the latent heat of crystallization of these near-eutectic compositions (Huber et al. 2009; Gelman et al. 2013; Caricchi and Blundy 2015), potentially permitting crystallization and microstructural development to occur during prolonged periods at near-constant temperature.

Ostwald ripening is slow in systems in which attachment kinetics are entirely controlling (leading to facetted grains, German et al. 2009). Observed rates of ripening are sufficiently slow for quartz (Cabane et al. 2001) and plagioclase (Cabane et al. 2005), both of which grow by a birth-and-spread mechanism (Kirkpatrick et al. 1976; Bindeman 2003), that Ostwald ripening, even on time-scales relevant to large silicic crystal mushes (10^5^ years), is not significant for grains larger than a few tens of microns (Cabane et al. 2005).

The likelihood that any ripening will result in extraction of grains from a mush depends on the number of contacts for each grain (Wolfsdorf-Brenner et al. 1999), together with their area. For a given number of contacts, the total contact area and ease of extraction will be lowest for texturally equilibrated systems with a low liquid–solid–solid dihedral angle (e.g., Niemi and Courtney 1983).

**Fig. 4:**
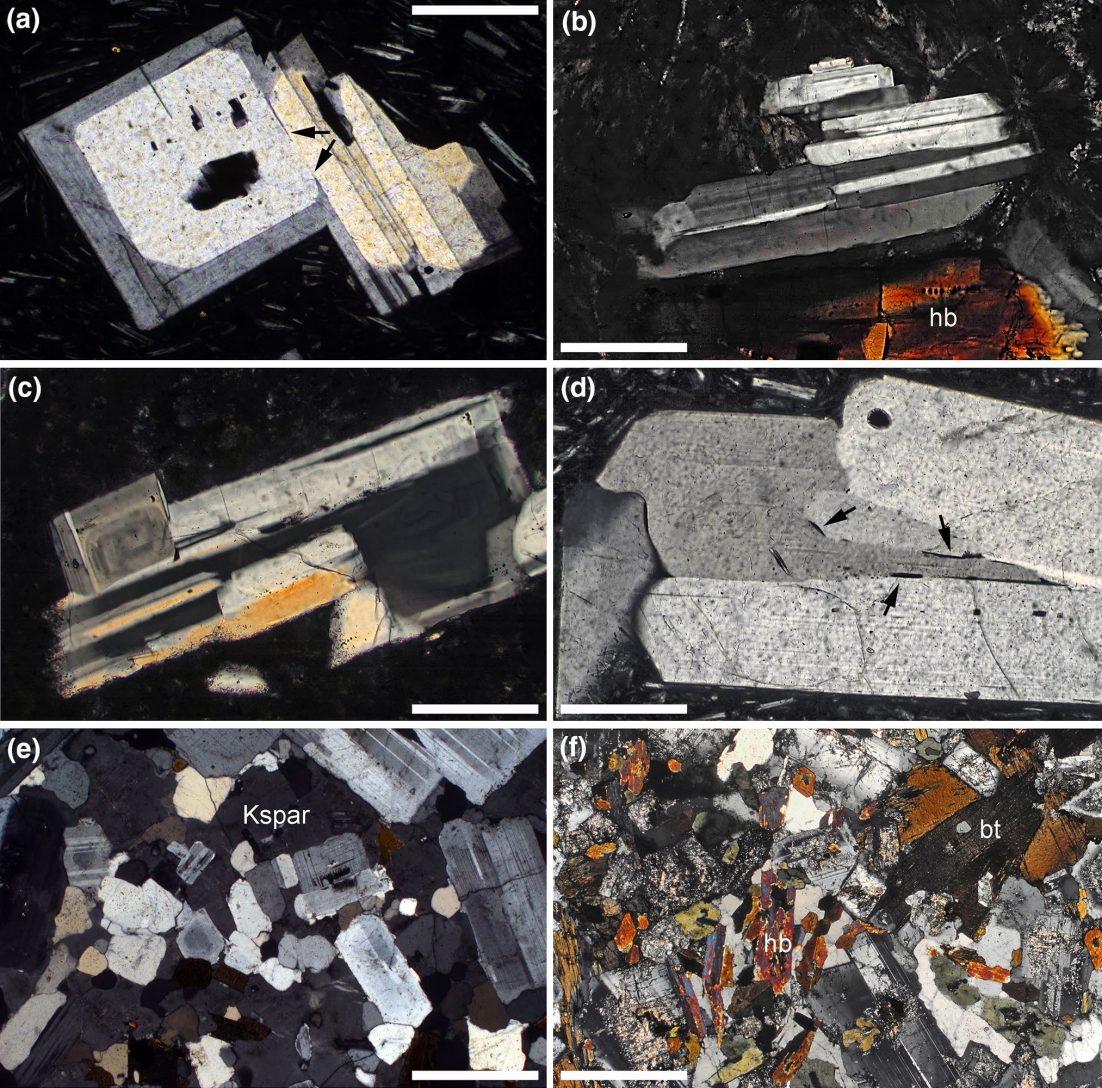
Photomicrographs under crossed polars. Sample numbers that are purely numerical refer to the catalogue number for the Harker Collection, Sedgwick Museum, University of Cambridge. **a** Plagioclase cluster in andesite erupted from the Kameni Islands, Santorini. Sample K0-5c (Martin et al. 2006). Note the two stages of growth discernible from the birefringence colours. The central parts of the two grains are rounded, with low dihedral angles developed at the junction (arrowed), indicative of growth rates commensurate with rates of textural equilibration, whereas the second (more rapid) stage of growth resulted in well-facetted grains and the development of a large contact area. Scale bar is 200 μm long. **b** Hornblende (hb) rhyodacite, Cape Akrotiri, Santorini, containing sintered crystal clots of plagioclase with large contact areas between individual grains. Sample 104988. Scale bar is 200 μm long. **c** Hornblende rhyodacite, Cape Akrotiri, Santorini. Note the complex aggregate containing at least five individual plagioclase grains that are well-sintered with large contact areas between individual grains. Sample 104988. Scale bar is 200 μm long. **d** Plagioclase cluster in andesite erupted from the Kameni Islands, Santorini. Sample K0-5c (Martin et al. 2006). While the contact area between the grains is large, small lenses of melt (arrowed) are still present locally. Scale bar is 200 μm long. **e** Bonsall Tonalite, Fallbrook, S. California Batholith, with crystal frameworks made of well-sintered, low aspect ratio, plagioclase enclosed and preserved in oikocrysts of K-feldspar (Kspar). Note the crystallographic preferred orientation of many grain pairs, with parallel (010) faces, and the large contact areas between grains. Sample 58219. Scale bar is 1 mm long. **f** Diorite. Scree above Cape Geology, Granite Harbour, South Victoria Land, Antarctica. Oikocrysts of K-feldspar enclose abundant framework-forming euhedral plagioclase and hornblende (hb). Biotite (bt) is a later-crystallising phase. Sample 81482. Scale bar is 1 mm long

A close approach to textural equilibrium in melt-bearing crustal rocks is rare: although melt-solid–solid dihedral angles may attain the equilibrium value, it is rare for the grains to achieve the lowest energy shape (Holness et al. 2005a, 2012). The contact area between grains in a silicate mush is thus not controlled by dihedral angle. Instead, synneusis of facetted silicate mineral grains commonly results in large contact areas, as observed for quartz (Beane and Wiebe 2012; Graeter et al. 2015), olivine (Schwindinger and Anderson 1989), and plagioclase (Vance 1969) (Fig. 4a–d). As an aside, the commonly observed presence of crystal clusters joined on large contact areas, such as seen in porphyritic andesites for example (Fig. 4), may be a sampling artifact, in that these joins are the hardest to break and, therefore, are the most likely to survive mush disaggregation associated with melt extraction and eruption. Even heterogeneous nucleation, as is common for plagioclase (Kirkpatrick 1977), results in frameworks characterised by large contact areas. It is highly unlikely that Ostwald ripening could act to free particles with such a large area of contact.

A further consideration is that particles in a crystal mush held at constant temperature may undergo grain growth by coalescence as well as Ostwald ripening (e.g., Schiavi et al. 2009). Coalescence prevents grain extraction and is promoted by the abundance of grain contacts in a mush with a high solid volume fraction [particularly if grains are joined by low-angle grain boundaries, e.g., Schiavi et al. (2009)], while Ostwald ripening is reduced in importance by the low solid–liquid surface area over which solution-reprecipitation can occur (Kipphut et al. 1988). Thus, as particles pack more closely, the likelihood of particle extraction by Ostwald ripening decreases.

In summary, micro-settling is an insignificant process in the extraction of melts from silicic mushes. The time-scale is insufficient to permit Ostwald ripening of particles other than those < 10 μm and particle extraction is likely to be sufficiently rare that it cannot contribute to melt segregation. Particle extraction at a free surface is, however, possible although likely to be unimportant. Since such a free surface would underlie a body of liquid-rich magma, extraction would only occur for buoyant grains which would detach from the mushy layer and rise into the magma, thus having the opposite effect to segregation.

## Hindered settling

Hindered settling of particles in a liquid occurs when the liquid fraction is sufficiently high that the particles are free to move relative to each other, but at particle concentrations sufficiently high to create fluid dynamical interactions between them. It occurs at particle fractions greater than a few vol.% (Happel and Brenner 1965) and leads to a significant decrease in the rate of settling compared to that of a single isolated particle. The calculations presented by Bachmann and Bergantz (2004) considered only the relationship between settling velocity and crystal fraction for systems in which the interactions between the particles are purely fluid dynamical (e.g., the effects on other particles of the upwards flow generated by the settling of nearby particles): this approach has been followed by subsequent studies (e.g., Faroughi and Huber 2015; Lee et al. 2016).

Here, for illustration, we turn again to the liquid-phase sintering literature and the pertinent observation that the volume fraction of the skeleton formed by settling particles is determined by the density difference between the solids and liquid (the LPS process generally involves only equant particles, so the undoubted effects of grain shape on skeleton formation are not relevant). Higher packings occur in systems in which the solid particles are relatively dense (Heaney et al. 1995). The explanation offered by Heaney et al. (1995) is that when the density difference between solid and liquid is small, the lower settling velocities allow time for sinter bonds to form between particles, forming loose clusters that then do not pack efficiently when they encounter other clusters. This leads to the formation of a particle framework at a lower packing density than for those formed of non-cohesive particles (Heaney et al. 1995). This explanation is highly germane to our understanding of silicate systems.

The effect of particle clustering on packing fraction can be marked. The random loose packing of cohesionless monodisperse spheres (defined as the loosest, mechanically stable packing state) comprises 56–54 vol.% solids (Onoda and Liniger 1990; Ciamarra and Coniglio 2008; Zamponi 2008; Farrell et al. 2010), although more efficient packings are possible for polydisperse spheres (Epstein and Young 1962; Jerram et al. 2003). However, the efficiency of random loose packing depends on particle cohesion: for strongly cohesive particles such as sintered aggregates of silicate minerals, a mechanically stable distribution can be achieved at volume fractions < 55% (Dong et al. 2006; Yang et al. 2007), and the presence of highly non-spherical, loose clustered chains will reduce this still further (Campbell 1978; Jerram et al. 2003). Random loose packing is also dependent on the viscosity of the liquid, with more open packings expected for particles in a viscous liquid (Delaney et al. 2011).

The abundant evidence for grain clustering via synneusis in magmatic systems (e.g., Vance 1969; Schwindinger and Anderson 1989; Schwindinger 1999; Nespolo and Ferraris 2004; Beane and Wiebe 2012; Graeter et al. 2015; Holness et al. 2017a), particularly in magmas in which there is some element of shearing (Schwindinger 1999) or in which particles are kept suspended by convection (Holness et al. 2017a), suggests that the assumption of purely fluid dynamical interactions between the settling particles is as inappropriate for magmatic systems as it is for sintered compacts. Furthermore, if settling occurs on time-scales commensurate with that of cooling, new particles will nucleate, either as primocrysts or as interstitial grains: nucleation will almost certainly be heterogeneous, potentially increasing cohesion between the settling particles.

The formation of a framework at low solid fraction is likely to be particularly important in silicic systems, particularly for feldspars which not only have a small density difference with the liquid, and thus a slow settling velocity, but also form non-equant tabular grains. Frameworks of plagioclase form in thick flood basalt flows at only ~ 25 vol.% solids (Philpotts et al. 1999; Philpotts and Dickson 2000).

The solid volume fractions used in the calculations of hindered settling rates by Bachmann and Bergantz (2004) extend to 50–60 vol.%, but the above discussion demonstrates that by this point in a silicate mush there will certainly be a cohesive framework. Even greater particle concentrations are suggested by Lee and Morton (2015) who argue, on the basis of geochemistry, that the cumulate residua from which highly silicic magmas have segregated (by hindered settling) are represented by tonalitic plutons containing only 20–30 vol.% interstitial liquid. It is extremely unlikely that such low porosities could be created by hindered settling of non-cohesive particles in a system with a plagioclase-rich liquidus assemblage.

The process of aggregation of initially isolated grains by synneusis followed by sintering can be detected by characteristic compositional zoning patterns (Fig. 4a; Schwindinger and Anderson 1989; Jerram et al. 2003; Beane and Wiebe 2012; Graeter et al. 2015), similar sizes of grains within a cluster (Schwindinger and Anderson 1989), differences in composition between grains within a cluster (Schwindinger and Anderson 1989), and a preferred relative orientation between adjacent grains due either to the greater likelihood of bonding at low energy grain boundaries (Fig. 4b–d; Vance 1969; Beane and Wiebe 2012; Graeter et al. 2015), or to the development of preferred orientations in a flow (Schwindinger 1999). Although many of these grain boundaries appear to be melt-free (Fig. 4a–c), some preserve evidence of incomplete melt expulsion during sintering (Fig. 4d), Future work should be aimed at detecting the microstructural record of framework formation in fully solidified plutons that are thought to have been the source of rhyolitic liquids, concentrating on the minerals that are on the liquidus in the early stages (since the later arriving minerals are likely to nucleate and crystallise at a stage post-dating that of hindered settling). Examination of oikocrysts is likely to be productive, since they commonly preserve an early state of mush development (Fig. 4e, f, e.g. Higgins 1991, 1998; Hunter 1996).

In summary, sintering results in the formation of mechanically coherent frameworks at solid fractions as low as a few tens of percent: these frameworks can only continue to expel liquid if they undergo either mechanical disruption or viscous deformation. Calculations of two-phase flow and segregation rates assuming hindered settling (e.g., Faroughi and Huber 2015) must take into account this early framework formation.

## Compaction

The significance of gravitationally-driven viscous compaction in gabbroic systems has recently been addressed by Holness et al. (2017b). Compaction, driven by the loading exerted by the crystal mush itself, is commonly cited as the mechanism by which accumulates are formed, in which the amount of residual liquid calculated from bulk compositions may be as low as a few vol.% (e.g. Sparks et al. 1985; Meurer and Boudreau 1998; McKenzie 2011). Holness et al. (2017b) argued that viscous compaction leaves a microstructural signature, which is clearly discernible if the deformation occurred by dislocation creep but may be harder to detect if the mush compacted by dissolution–reprecipitation. A consideration of the microstructures in the Skaergaard intrusion of East Greenland demonstrated the insignificance of such signatures, leading Holness et al. (2017b) to conclude that viscous compaction was not an important mechanism operating in these gabbroic cumulates.

Viscous compaction is potentially less important in silicic systems compared to mafic systems (McKenzie 1985; Miller et al. 1988), both because the former do not contain significant quantities of the dense minerals such as oxides which result in a significant gravitational loading of oxide-rich mafic mushes (e.g., Holness et al. 2017b), and because the viscosity of interstitial liquids in silicic mushes is generally higher than that in mafic systems. However, a critical assessment of its significance in silicic systems is complicated. First, silicic intrusions commonly form in tectonically active areas, so an assessment of the extent of gravitationally-driven viscous compaction needs not only to “see through” any superimposed sub-solidus deformation but also to detect how much of the super-solidus deformation was internally generated by gravitational collapse and how much was externally imposed by regional stress fields (e.g., Paterson et al. 1989, 1998; Clemens and Mawer 1992; Vernon and Paterson 1993; Sawyer 2000; Garibaldi et al. 2017). Second, the amount of compaction required to generate a large eruptible body of crystal-poor rhyolite from an even larger body of crystal mush might be small (e.g., Lee and Morton 2015), leaving a barely discernible microstructural record.

### Microstructural indicators of compaction in silicic crystal mushes

In contrast to the previous two mechanisms for melt segregation, the detection of compaction necessitates the decoding of fabrics in silicic plutonic rocks [see Paterson et al. (1989) and (1998) and Vernon (2000) for comprehensive reviews of the literature]. Fabric formation is a continuum, with the earliest forming as the melt is sufficiently abundant to allow grains to move past each other, and the latest forming when all melt has solidified. The change from suspension flow to grain-supported flow occurs when the melt fraction drops to 20–40 vol.% (Paterson et al. 1998) with the precise fraction required for the change dependent on the porosity at which mechanically coherent frameworks form, itself a function of grain shape (Picard et al. 2013). Once the crystals have formed a framework, we enter the realm of “submagmatic flow” (Paterson et al. 1989), and this realm is where viscous compaction takes place.

The mechanisms by which deformation occurs during submagmatic flow are controlled by the amount and spatial distribution of the melt. In rocks containing very little melt, deformation is primarily by dislocation creep (Cooper and Kohlstedt 1984; Dell’Angelo et al. 1987), whereas for mushes containing abundant melt, a range of processes are possible such as melt-assisted grain boundary sliding, melt-assisted diffusion creep (Dell’Angelo et al. 1987), contact-melting assisted grain boundary migration (Park and Means 1996), strain partitioning into melt-rich zones, and grain rotation. The operation of some of these may be difficult to infer from microstructures, particularly since the assembly of significant volumes of crystal-free rhyolitic liquid may require only limited compaction of a large body of silicic mush: Rosenberg (2001) offers a comprehensive review of criteria which can be used to identify syn-magmatic deformation and the following builds on his work.

**Fig. 5:**
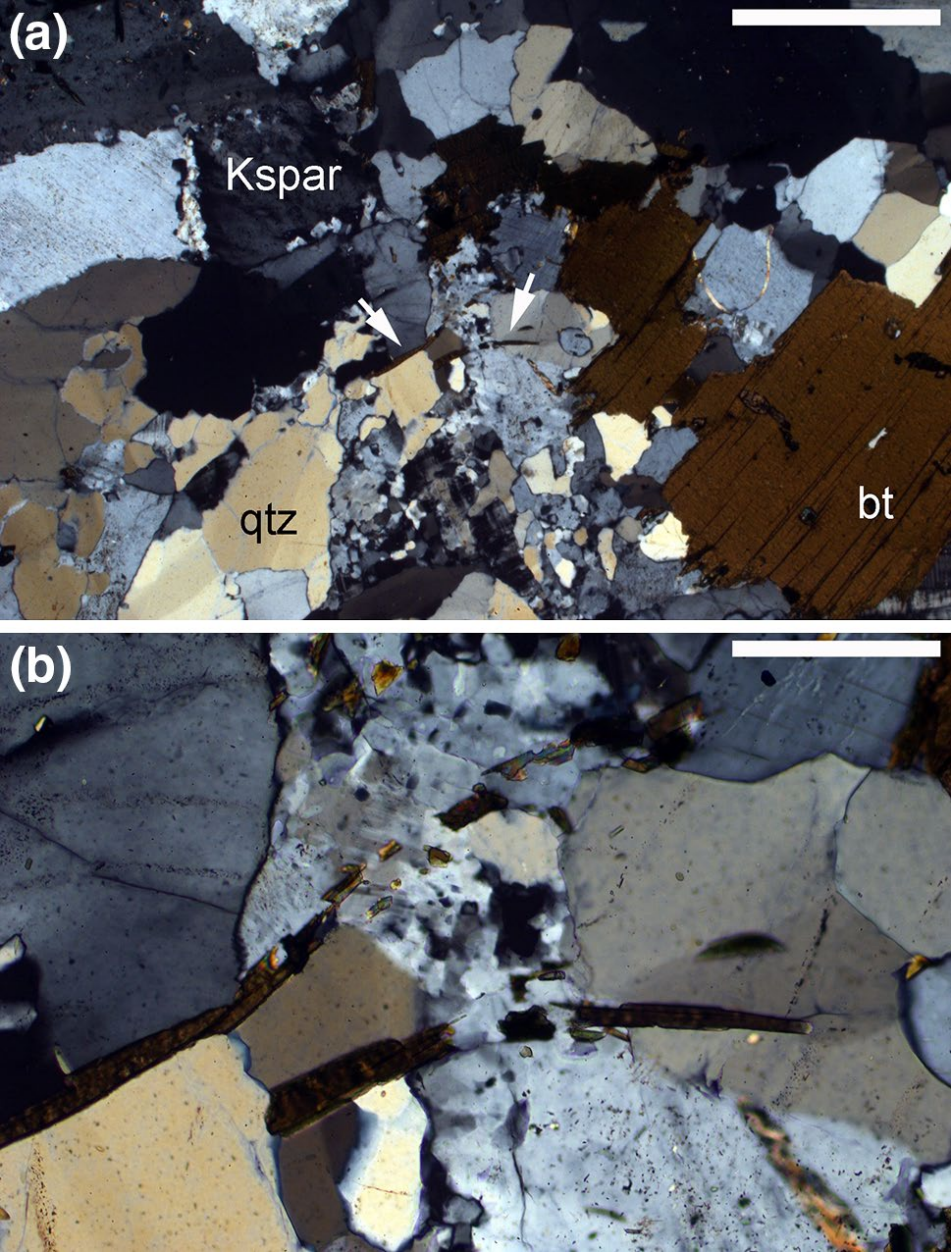
Photomicrographs under crossed polars of the Woodson Mountain granodiorite, Rainbow, S. California Batholith (sample 58260 from the Harker Collection, Sedgwick Museum, University of Cambridge). **a** Zone of fine-grained quartz and feldspar runs up the centre of the field of view, separating large (presumed early crystallising) grains of quartz (qtz) and K-feldspar (Kspar). The arrows show the location of a pair of thin biotite laths that have been broken by the formation of this syn-magmatic vein. Scale bar is 1 mm long. **b** Close-up of the region containing the broken biotite in **a**. Scale bar is 200 μm long

**Fig. 6:**
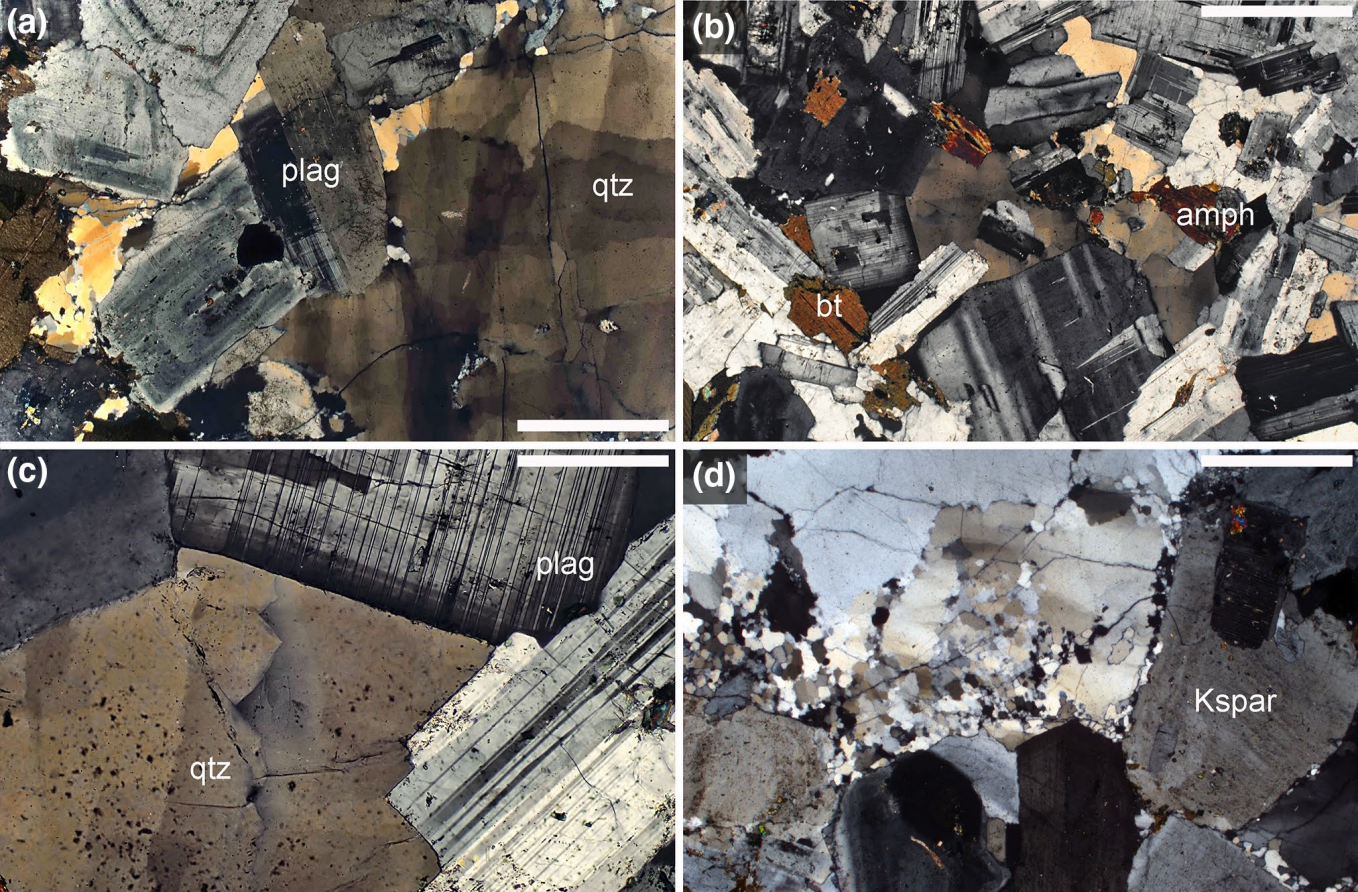
Photomicrographs under crossed polars. Sample numbers refer to the Harker Collection catalogue, Sedgwick Museum, University of Cambridge. **a** Domenigoni granodiorite, Domenigoni Valley, S. California batholith. Euhedral plagioclase grains (plag) exhibiting concentric composition growth zoning are cemented by quartz (qtz) with undulose extinction. The plagioclase shows no signs of dislocation creep. Sample 58224. Scale bar is 1 mm long. **b** Hornblende-biotite-tonalite, Yosemite National Park (near Tiaga Pass). This rock comprises a well-formed framework of euhedral facetted plagioclase grains, cemented by interstitial quartz. The only sign of dislocation creep is the undulose extinction in the quartz. Sample 71511. Scale bar is 1 mm long. **c** Close-up of a region of **b**, demonstrating the undulose extinction in the quartz, while the zoned plagioclase bounding the interstitial quartz has no sign of dislocation creep and a well-defined facetted growth surface. Sample 71511. Scale bar is 200 mm long. **d** Domenigoni granodiorite, Domenigoni Valley, S. California batholith. Note the extensively developed fine-grained regions in the interstitial quartz, indicative of dynamic recrystallization during dislocation creep. Recrystallization is particularly concentrated at boundaries with the (undeformed) plagioclase. Sample 58224. Scale bar is 1 mm long

There is sparse reported evidence for syn-magmatic deformation by dislocation creep in silicic plutonic rocks, which is straightforwardly demonstrated if deformed grains are cemented by later-crystallising undeformed grains (Bouchez et al. 1992) (Fig. 5). Since quartz and biotite are both weak in comparison to feldspars and amphibole, early crystallising quartz and biotite should be bellwethers for syn-magmatic deformation. However, the most commonly observed situation in tonalites is that undulose extinction is restricted to interstitial quartz, with the framework-forming euhedral plagioclase grains apparently entirely undeformed (Fig. 6a–d): deformation must have occurred in the latest stages of, or post-dated, solidification. In such rocks, dynamic recrystallization is commonly initiated at quartz-plagioclase grain boundaries (Fig. 6d). The commonly observed irregularity of boundaries between feldspar and quartz (e.g., Fig. 6a, although compared with Fig. 6d) is not due to dynamic recrystallization but is more likely a consequence of localized simultaneous growth of the two phases during solidification.

The dearth of published evidence for syn-magmatic dislocation creep suggests that any viscous compaction of silicic mushes must have occurred at sufficiently low strain rates (and/or high enough melt fractions) to enable creep by diffusion-controlled processes involving dissolution–reprecipitation [or melt-assisted diffusion creep (Dell’Angelo et al. 1987)] or grain boundary sliding (Rosenberg 2001).

Grain boundary sliding in rocks with a low melt fraction and irregularly shaped grains creates localized stress concentrations sufficient to promote detectable localized plastic deformation or recrystallization (Vernon 2000). Its agency may, however, be impossible to distinguish in fully solidified microstructures which underwent grain boundary sliding at high melt fractions (Park and Means 1996; Rosenberg 2001). In contrast, dissolution–reprecipitation does leave a recognizable microstructural signature.

The efficacy of melt-assisted diffusion creep and creep by dissolution–reprecipitation is determined by the amount of grain boundary wetted by melt (e.g., compare the immediately post-sintering contact area of the two plagioclase grains in Fig. 4a, with that immediately prior to eruption, as inferred from zoning patterns). Microstructures characteristic of creep by dissolution–reprecipitation mechanisms have low dislocation densities (although densities may be locally high at grain–grain contacts), with irregularly shaped, embayed, and scalloped grains (Dell’Angelo et al. 1987).

During viscous compaction by dissolution/reprecipitation, fabrics resulting from magma flow (e.g., Paterson et al. 1998; Park and Means 1996), and defined by a shape preferred orientation (SPO), might be enhanced or destroyed, depending on the relative orientation of the primary fabric and the compaction stress. Park and Means (1996) argue that were the mush to have no primary fabric, dissolution/reprecipitation would create an SPO that would bear no relationship with the crystallographic orientation of the grains, particularly if deformation is associated with grain boundary sliding. This feature is observed in metamorphic rocks deformed by dissolution–precipitation creep (Stöckhert et al. 1999). However, a crystallographic preferred orientation (CPO) could be created in conjunction with an SPO if there is anisotropy of dissolution as is shown, for example, by plagioclase (Heidelbach et al. 2000; Arvidson et al. 2004) and K-feldspar (Menegon et al. 2008).

The detection of viscous compaction by a dissolution/reprecipitation mechanism requires a systematic search for sources and sinks (Wassmann and Stöckhert 2013), and the identification of spatial relationships consistent with uniaxial deformation. Sources can be identified on the basis of truncation of objects of known or presumed original shape, such as euhedral facetted grains (although this will not work for bulk compositions at or close to final eutectics, since the simultaneous growth of all phases leads to early impingement and irregular grain shapes), the truncation of compositional zoning formed during primary growth (e.g., Rosenberg 2001; Vernon and Collins 2011), and the passive localized enrichment of inclusions of the second-phase particles that are undergoing less efficient dissolution. Although compositional zoning formed during primary crystallization may be truncated during continued growth after grain impingement, the record of viscous compaction will be preserved in a preferred orientation of the zone-truncating grain boundaries. Similarly, compaction will result in a preferred orientation of indented boundaries that result from dissolution. Zone truncation due to dissolution–precipitation creep in high-grade metamorphic rocks has been detected using CL in quartz (Stöckhert et al. 1999) and by imaging major element zoning in BSE images (e.g., Wintsch and Yi 2002; Stokes et al. 2012; Wassmann and Stöckhert 2013). No comparable studies have been undertaken of silicic plutons.

Sources are generally represented by features at interfaces between crystals, particularly those between two grains of different phases: in contrast, sinks tend to be more localized (Wassmann and Stöckhert 2013). Sinks can be identified by microstructural features indicating crystallization in an open melt-filled cavity (e.g., Fig. 5), overgrowths on mineral grains resulting in elongation perpendicular to the maximum compressive stress, and strain shadows filled with late-crystallising minerals. Cooper and Kohlstedt (1984) argue that facetted grains facing into large melt-filled pores are indicative of dissolution/reprecipitation, and this feature is indeed very common in tonalites (e.g. Fig. 6a–c). However, Cooper and Kohlstedt (1984) base this argument on microstructures developed in experiments run at a constant temperature: in mushes undergoing active crystallization; the development of planar facets is simply indicative of unimpeded interface-controlled growth and cannot be used to argue for dissolution/reprecipitation creep.

**Fig. 7:**
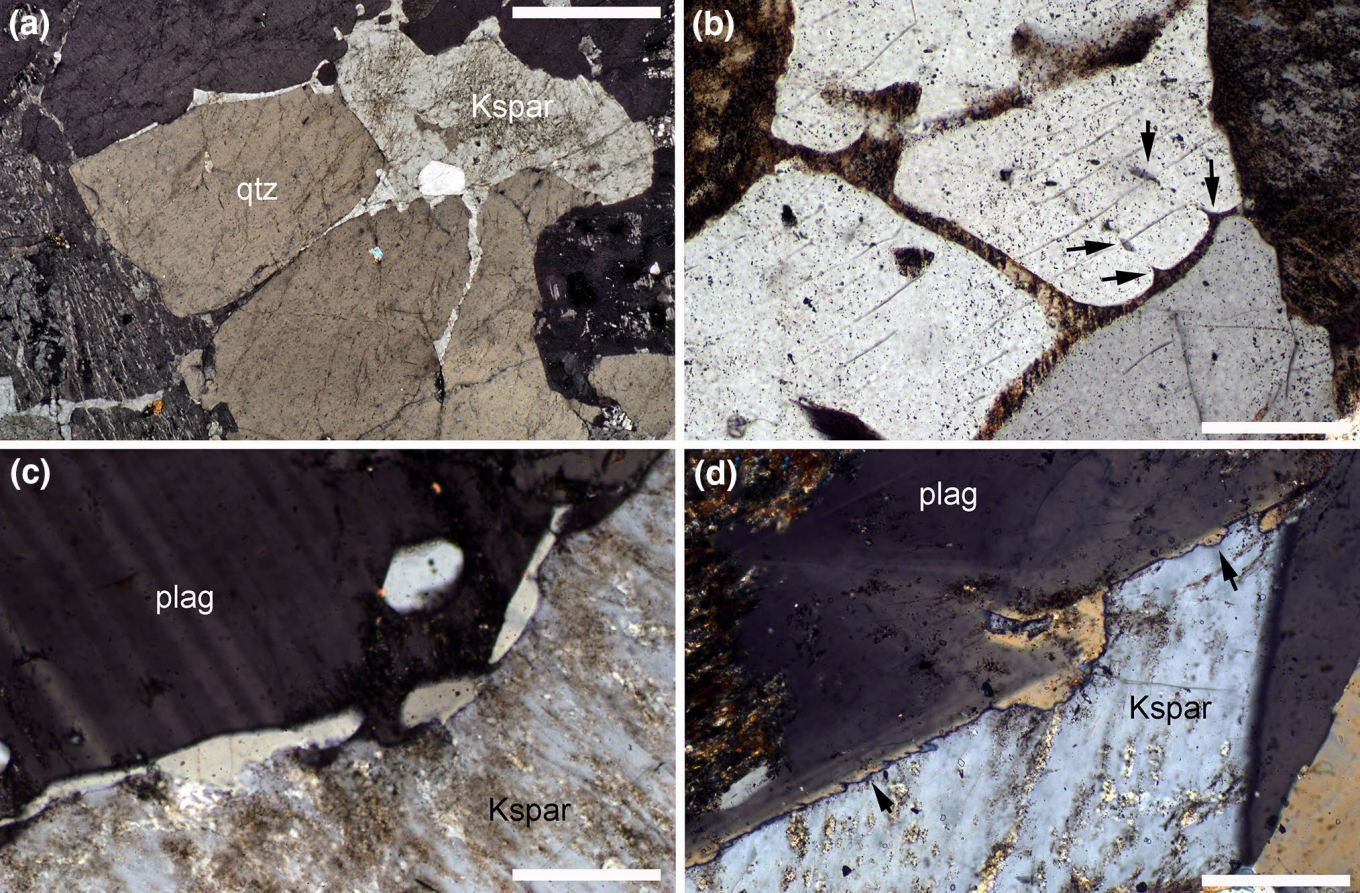
Photomicrographs under crossed polars. **a** Hornblende granite, Marsco, Skye. Note the highly attenuated apophyses of K-feldspar (Kspar) extending down grain boundaries in the adjacent quartz (qtz). Using migmatite microstructures as an analogue, these form during the last stages of solidification and show where the last porosity was situated. Sample 135634 of the Harker Collection, Sedgwick Museum, University of Cambridge. Scale bar is 1 mm long. **b** Granite from Arran, Scotland. Note the cuspate extension of K-feldspar that extend along quartz grain boundaries. In places, these form cuspate shapes that align with small lenses within single grains of quartz (arrowed). Such features are suggestive of the existence of a quartz–quartz grain boundary that has been annihilated by grain coalescence. Scale bar is 200 μm long. **c** Biotite granite from Shap Quarry, Westmorland, UK. Note the rounded quartz lenses on the grain boundary separating large grains of plagioclase and K-feldspar. The quartz most likely crystallized from late-stage melt. Sample 28483 (Harker Collection, Sedgwick Museum, University of Cambridge). Scale bar is 200 μm long. **d** Biotite granite from Shap Quarry, Westmorland, UK. A compositionally zoned plagioclase grain with an irregular growth face (marked by the compositional zoning) is separated from the adjacent grain of K-feldspar by a continuous grain of quartz (arrowed) which pseudomorphs the residual porosity. Sample 28481 (Harker Collection, Sedgwick Museum, University of Cambridge). Scale bar is 200 μm long

Perhaps, the most convincing evidence for sinks is the presence of late-stage magmatic minerals in positions indicative of migration of residual melt into lower pressure sites, such as pressure shadows around large grains (Paterson et al. 1989). An example of this is the identification of micro-fractures in individual grains of early crystallising phases sealed with late-stage magmatic minerals (e.g., sodic plagioclase or quartz) (Bouchez et al. 1992; Berger et al. 2017), although neither study offered a systematic examination of the orientation of such fractures that could be used as an indicator of the stress field and, therefore, in support of viscous compaction. Late-stage porosity is commonly pseudomorphed by single grains of quartz or feldspar, forming generally cuspate interstitial grains with low dihedral angles [Fig. 7; e.g., Holness and Sawyer (2008) and references therein]. If solidification occurred during compaction, such pseudomorphs would have a preferred orientation of elongation parallel to the direction of maximum compression.

Although there is convincing microstructural evidence of creep by dissolution–precipitation preserved in high-grade metamorphic rocks (Stöckhert et al. 1999; Wintsch and Yi 2002; Stokes et al. 2012; Wassmann and Stöckhert 2013), and in experimental studies of melt-bearing silicate mushes (Cooper and Kohlstedt 1984; Dell’Angelo et al. 1987), no microstructural evidence has yet been reported that supports the operation of this deformation mechanism during gravitationally-driven viscous compaction of silica-rich mushes.

## Discussion

The foregoing consideration of the three melt segregation mechanisms initially suggested by Bachmann and Bergantz (2004), all essentially driven by differences in buoyancy between solids and liquid, shows that micro-settling is not viable, hindered settling can only operate as modelled in the literature at very low particle concentrations, and no convincing microstructural evidence has yet been provided to support the widespread, gravitationally-driven, viscous compaction of silica-rich mushes. If we discard the three Bachmann and Bergantz (2004) mechanisms, how else might large volumes of crystal-poor silicic liquid be assembled? In the following sections, alternative mechanisms are discussed.

### Magma recharge

The arrival of batches of hotter magma has been suggested as an important process that results in the thermal rejuvenation of mush, leading to the mobilization of interstitial liquid (Charlier et al. 2007; Bachmann et al. 2007; Bergantz et al. 2015; Paterson et al. 2016; Sato et al. 2017; Sliwinski et al. 2017). Evidence of thermal rejuvenation is provided by the corrosion of low-temperature minerals (such as quartz and feldspar), mineral zoning recording increasing temperatures (Allan et al. 2013; Barker et al. 2016), whole-rock homogeneity, and field-scale features such as enclaves and evidence of erosion [see Bachmann and Huber (2016) for a recent review].

### Gas filter-pressing

Exsolution of gas from the interstitial liquid in a rigid crystal framework drives liquid through the mush (Anderson et al. 1984; Bacon 1986), with potential to extract large eruptible bodies of crystal-poor rhyolite from a crystal mush (Sisson and Bacon 1999). For gas filter-pressing to be effective, magma H_2_O contents should be high and solidification must occur in the shallow crust. In lava flows, vesicles lined with evolved liquid (Anderson et al. 1984), the distribution of evolved glass in and around crystalline inclusions (Bacon 1986), and vertical vesicular pipes (Fowler et al. 2015) preserve abundant evidence that this process is important at least immediately prior to, during, and after eruption, but what evidence would be preserved in a solidified crystal mush that had lost interstitial liquid in this manner?

Hartung et al. (2017) suggest, on the basis of the coincidence between the crystallinity at which melt extraction occurred and the achievement of water saturation, that gas filter-pressing may have been important in melt extraction. Gas filter-pressing requires a rigid crystal framework, and results in the creation of vesicles and vugs. The presence of abundant miarolitic cavities in the solidified plutonic source rocks of rhyolitic magmas would thus be expected, unless the rigid crystal framework compacted, expelling the exsolved volatiles. In the latter case, gas filter-pressing could, therefore, be argued for using a combination of geochemical arguments (e.g., Hartung et al. 2017) and microstructural evidence for compaction.

### Segregation driven by external stress

Several recent contributions (e.g., Nasipuri et al. 2011; Allan et al. 2013, 2017; Webber et al. 2015; Garibaldi et al. 2017) have suggested that the segregation of rhyolitic melts from a crystal mush can be enhanced by an external stress field. This is not a new idea: the requirement for deformation-driven melt segregation has long been recognized by those working on anatectic regions of the crust (reviewed by Rosenberg 2001), with a commonly observed linkage and feedbacks between regional tectonics, migmatite segregation, and granite emplacement (e.g., Brown and Solar 1998). Similarly, abundant field evidence shows that tearing and slumping caused by gravitational collapse of crystal mushes on the roof and walls of mafic intrusions results in melt segregation (e.g., Philpotts et al. 1996; Marsh 1996; Philpotts and Dickson 2002; Humphreys and Holness 2010).

The effect of external stress fields on melt segregation has been extensively explored using experimental charges undergoing simple shear (e.g., Holtzman et al. 2003, Holtzman and Kohlstedt, 2007; Rosenberg and Handy 2000) [although see Rosenberg and Handy (2001) and Kohlstedt and Holtzman (2009) for reviews of work involving pure shear applicable to gravitationally-driven compaction]. The strong coupling between melt topology and mush rheology during shearing leads to effective segregation via the creation of melt-bearing channels oriented at a low angle to the shear plane and antithetic to the shear direction (Kohlstedt and Holtzman 2009; Rosenberg and Handy 2000, 2001; Rosenberg and Riller 2000; Sawyer 2000; Nasipuri et al. 2011; Allan et al. 2013). These experimental results are supported by field evidence of rapid and efficient melt segregation during non-coaxial deformation of partially molten rocks (e.g., Nasipuri et al. 2011; Berger et al. 2017), with melt migration into dilatant structures such as boudin necks and shear bands within a decade or 2 decades, driven by pressure gradients that set up by heterogeneous deformation (Sawyer 1991).

An important question, however, is the extent to which these experimental studies and field observations of migmatites are relevant to melt extraction from solidifying tonalitic mushes. A critical difference is that the segregation of melts from migmatites occurs during an increase in temperature (i.e., during melting), whereas the segregation of evolved interstitial liquid from a crystal mush occurs during a decrease in temperature (i.e., during solidification), essentially encapsulating the dichotomy concerning rhyolites as either sourced directly from melting source rocks or as evolved interstitial liquids expelled from a crystal mush.

The grain-scale distribution of liquid in melting and solidifying systems is not the same, and this difference introduces poorly-understood discrepancies in the behavior of the two types of system. For example, Sawyer (2000) pointed out that residual melt in solidifying granites is concentrated in films separating euhedral plagioclase grains, in contrast to irregular melt films separating rounded grains of reacting phases in migmatites (e.g., Holness et al. 2005b). On a larger scale, the melt distribution in migmatites shows evidence of organization into discrete channels and these outcrop-scale features are not common in silicic plutons. Finally, the reactions responsible for much crustal melting involve a positive volume change (Clemens and Mawer 1992), increasing permeability by internally-generated hydrofracture (e.g., Clemens and Mawer 1992; Connolly et al. 1997) which is not likely in solidifying systems.

If it is, indeed, the case that an externally-imposed stress field is required to generate large volumes of crystal-poor rhyolite, such eruptions should be concentrated in tectonically active regions. The Taupo Volcanic Zone of New Zealand fits the bill, with Allan et al. (2013, 2017) arguing strongly that regional deformation played a key role in generating large bodies of crystal-poor rhyolite. Similarly, the Long Valley volcanic field in California (which includes the Bishop Tuff) is contemporaneous with regional trans-tensional strain (Hildreth 2004). There is also excellent field and geochemical evidence for efficient melt segregation in actively deforming compressional environments such as subduction zones (Schaen et al. 2017), suggesting that the precise nature of the external stress field may not be important, simply that melt segregation will be enhanced during active deformation.

## Conclusions

In this contribution, I have argued that we need to reconsider the commonly postulated mechanisms, all essentially driven by internally-generated buoyancy forces, by which large volumes of crystal-poor rhyolitic magma can be extracted from their tonalitic mushy nurseries. Micro-settling is not viable as a process in either metals or silicate mushes, and hindered settling as generally modelled in the melt segregation literature is only applicable for crystal-poor systems. No microstructural evidence has yet been reported that supports widespread gravitationally-driven viscous compaction of crystal mushes by either dislocation creep or creep facilitated by diffusion: clearly, we should concentrate efforts on searching for the sort of supporting evidence outlined above to demonstrate that compaction is, indeed, an important mechanism operating in silicic mushes.

A complete understanding of the rates and mechanisms by which interstitial melt might segregate from a solidifying crystal mush essentially requires a better understanding of how crystal mushes behave. Future experimental work should be directed at studying the timing and rates of clustering during settling, the porosity of the resultant framework, and the rates and extent to which it collapses, particularly under shear. The time-scales of segregation in mushes undergoing deformation should be investigated to determine whether regional tectonic stresses are sufficient to create melt drainage networks on appropriate time-scales (e.g., Schoene et al. 2012). This information can then be combined with geochemical approaches to place better constraints on the source of crystal-poor rhyolites and differentiate between an origin in partially melted material or in solidifying crystal mushes.
